# “If donors woke up tomorrow and said we can't fund you, what would we do?” A health system dynamics analysis of implementation of PMTCT option B+ in Uganda

**DOI:** 10.1186/s12992-017-0272-2

**Published:** 2017-07-26

**Authors:** Tanya Doherty, Donnela Besada, Ameena Goga, Emmanuelle Daviaud, Sarah Rohde, Nika Raphaely

**Affiliations:** 1 Health Systems Research Unit, South African Medical Research Council, Cape Town, South Africa; 2 School of Public Health, University of the Western Cape, Cape Town, South Africa; 3 School of Public Health, University of the Witwatersrand, Johannesburg, South Africa; 4 Department of Paediatrics, University of Pretoria, Pretoria, South Africa; 5 Gender and Health Research Unit, South African Medical Research Council, Pretoria, South Africa

**Keywords:** HIV, Health systems, Option B +, Prevention of mother to child HIV transmission (PMTCT), Rapid appraisal

## Abstract

**Background:**

In October 2012 Uganda extended its prevention of mother to child HIV transmission (PMTCT) policy to Option B+, providing lifelong antiretroviral treatment for HIV positive pregnant and breastfeeding women. The rapid changes and adoptions of new PMTCT policies have not been accompanied by health systems research to explore health system preparedness to implement such programmes. The implementation of Option B+ provides many lessons which can inform the shift to ‘Universal Test and Treat’, a policy which many sub-Saharan African countries are preparing to adopt, despite fragile health systems.

**Methods:**

This qualitative study of PMTCT Option B+ implementation in Uganda three years following the policy adoption, uses the health system dynamics framework to explore the impacts of this programme on ten elements of the health system. Qualitative data were gathered through rapid appraisal during in-country field work. Key informant interviews and focus group discussions (FGDs) were undertaken with the Ministry of Health, implementing partners, multilateral agencies, district management teams, facility-based health workers and community cadres. A total of 82 individual interviews and 16 focus group discussions were completed. We conducted a simple manifest analysis, using the ten elements of a health system for grouping data into categories and themes.

**Results:**

Of the ten elements in the health system dynamics framework, context and resources (finances, infrastructure & supplies, and human resources) were the most influential in the implementation of Option B+ in Uganda. Support from international actors and implementing partners attempted to strengthen resources at district level, but had unintended consequences of creating dependence and uncertainty regarding sustainability.

**Conclusions:**

The health system dynamics framework offers a novel approach to analysis of the effects of implementation of a new policy on critical elements of the health system. Its emphasis on relationships between system elements, population and context is helpful in unpacking impacts of and reactions to pressures on the system, which adds value beyond some previous frameworks.

## Background

In Uganda, 790,000 women were living with HIV in 2015. HIV prevalence amongst pregnant women has declined from 30% in 1990 to 7% in 2012 [1], largely due to overseas development assistance (ODA) to scale up Uganda’s HIV/AIDS response [2]. The 2012 national AIDS spending assessment report shows that Uganda’s HIV response was financed by the Government of Uganda (12%), development partners (68%), and private sources including out-of-pocket expenditure (20%). The largest contributor to Uganda’s HIV/AIDS response is the United States President’s Emergency Plan for AIDS Relief (PEPFAR), which provided a consistent $324 million per year between 2011 and 2015 [3].

Globally, strategies to prevent mother to child HIV transmission (PMTCT) evolved progressively from single dose nevirapine prophylaxis in 2000 to the current recommendation that all pregnant and breastfeeding women, irrespective of CD4 count, should receive lifelong antiretroviral treatment (ART), known as Option B+ [4]. Modelled estimates predict this approach to have substantial clinical and programmatic advantages, including simplification of regimen, harmonisation with ART programmes, protection of future pregnancies from transmission and prevention of HIV transmission to serodiscordant partners [4]. However, insufficient consideration has been given to health systems’ readiness to absorb a new service requiring regular follow-up on a large section of the population for lifelong chronic treatment [5].

Malawi was the first country to implement Option B+ in 2011 as a pragmatic approach to improve PMTCT coverage, which sparked interest in the global public health community [6]. In 2012, the World Health Organization (WHO) released a programmatic update recommending Option B+ [4], and in September 2015, further guidance recommended extending lifelong ART to everyone living with HIV, widely termed ‘Universal Test and Treat’ (UTT) [7]. In 2012, soon after the WHO programmatic update was released, Uganda amended its PMTCT policy to Option B+, providing lifelong ART for HIV-infected pregnant and lactating women.

This rapid policy change was not accompanied by health systems research to understand health system preparedness, particularly regarding critical elements such as human resources for health, infrastructure and supplies, finances and service delivery. Early implementation lessons from Option B+ in eleven Elizabeth Glaser Pediatric AIDS Foundation (EGPAF) supported African countries including Uganda, [8] found that the required task-shifting had led to staffing pressures, especially on lower level cadres who are largely responsible for counselling and community follow up. The pressure of global commitments and the speed of policy change and scale-up without a simultaneous focus on health system strengthening, raises questions regarding the likelihood of this policy achieving its stated goal of eliminating vertical HIV transmission [9, 10].

HIV/AIDS is one of several health programme areas that experienced the introduction of new policies and interventions during the Millennium Development Goal (MDG) era. The emergence of a plethora of global health initiatives (GHIs) targeting specific diseases or programme areas (e.g. newborn health and malaria) with ambitious targets have prompted the establishment of parallel systems to achieve results within weak health systems [11, 12]. Although the impact of new policies and interventions on health systems has not received sufficient attention, evidence is accumulating that substantial investments into disease-specific programmes, such as rapid scale-up of HIV/AIDS services [13–15], despite having positive effects on the specific programmes, have led to little spill-over into system-wide benefits. Furthermore, research undertaken in several countries has found unintended negative consequences for other health programmes, such as reduced funding or loss of scarce personnel to better paying jobs in international organisations [16–19].

This paper presents findings from a rapid assessment of PMTCT Option B+ implementation in Uganda three years after policy adoption. Using the van Olmen et al. health system dynamics framework [20] it aims to explore the impacts of this policy shift on the health system. The van Olmen framework, an update of a previous WHO health system framework [21], is based on the premise that health systems are complex adaptive systems comprised of several elements that interact and respond to change. This framework was chosen as it includes elements, such as population and context not present in previous frameworks. This strengthens our understanding of interactions and dynamic relationships between elements of a health system and the context in which they are implemented.

## Methods

### Study design

The research was part of an evaluation of the Optimizing HIV Treatment Access (OHTA) initiative for pregnant and breastfeeding women. The initiative, funded by the governments of Sweden and Norway through the United Nations Children’s Fund (UNICEF), was undertaken in four countries (Malawi, Uganda, the Democratic Republic of Congo (DRC) and Côte d’Ivoire) between 2013 and 2017 in partnership with several IPs and their local agencies [22]. In Uganda, the OHTA initiative aimed to optimize implementation of Option B+, with particular focus on strengthening community-facility linkages and supporting routine monitoring to enhance retention in care.

This descriptive qualitative study [23] used rapid appraisal methodology [24] to explore the impact of PMTCT Option B+ implementation on the Uganda health system. Rapid Appraisal is an approach that draws on multiple evaluation methods and techniques to quickly, yet systematically, collect data when time in the field is limited and research findings are needed in a timely manner for decision-makers [24]. This methodology was chosen as it would provide findings in a short period of time which could inform planning for the completion of the OHTA initiative and handover of activities to the Ministry of Health and local implementing partners (IPs).

### Data collection

Qualitative data was collected from individual interviews and focus group discussions (FGDs) during country-level field work and through desk review (annual project reports from UNICEF and IPs, annual country reports to UNICEF, national PMTCT strategic plans and academic literature). A field visit lasting nineteen days was undertaken in June/July 2015 by a team of five researchers. Potential organisations and individuals for key informant interviews and FGDs were identified through the desk review process and were amended in collaboration with UNICEF headquarters and the Uganda UNICEF office.

Semi-structured interview guides were developed for each category of respondent (Ministry of Health, IPs, multilateral agencies, district management teams, facility-based and community health workers).

Each interview, conducted by one or more researchers, lasted between 30 min and one hour. Where necessary, a translator explained the research aim and consent process and translated the interview. Interviews were audio-recorded where permission was granted, and researchers took notes. Signed informed consent from literate participants, or recorded verbal consent from illiterate participants, was obtained.

The country visit included meetings in Kampala (Central region), followed by travel to nine districts in the Eastern, Northern and Western Regions for visits to district management teams and health facilities. All four regions of the country were included as the regions are geographically and culturally distinct, and the OHTA initiative provided support across all regions. Overall, 82 individual interviews and 16 FGDs were recorded and transcribed (Table 1). All interviewees were Ugandan except for some employees of multilateral agencies and IPs, who were citizens of other African countries.

**Table 1:** Summary of participants

Field work 29th June to 19th July 2015: Greater Kampala and 9 districts across three regions (Bugiri, Kamuli, Kaliro, Isingiro, Bushenyi, Ibanda, Moroto, Kotido, Abim).		
Type of interview	Participant category	Number of interviewees/ focus group discussion participants
Individual interviews	Implementing partners (EGPAF, M2 M, CUAMM, Baylor, STAR-EC)^a^	15
	Ministry of Health	6
	Multilateral agencies (UNICEF and WHO)	2
	District management	57
	Community-based health worker	2
Focus Group Discussions	Implementing partner	1 group with 3 participants
	Facility-based health workers	2 groups (average size 4 participants)
	Community-based health workers (village health team (VHT), lay counsellors, mentor mothers, community health workers, traditional leaders, expert clients, male champions, peer educators)	13 groups (average size 5 participants)

^a^ *EGPAF* Elizabeth Glaser Pediatric AIDS Foundation, *M2 M* Mothers2mothers, *CUAMM* Medici con l’Africa, *Baylor* Baylor College of Medicine, *STAR-EC* Strengthening HIV&AIDS and Tuberculosis Responses in East Central Uganda

### Data analysis

Audio-recorded interviews were transcribed and field notes summarized. This analysis was undertaken subsequent to the evaluation contracted by UNICEF. We used the van Olmen et al. [20] health system dynamics framework, consisting of ten elements (context, population, leadership and governance, infrastructure and supplies, human resources, knowledge and information, finances, service delivery, outcomes and goals) focusing on interactions between elements, as an analytic framework.

**Fig. 1:**
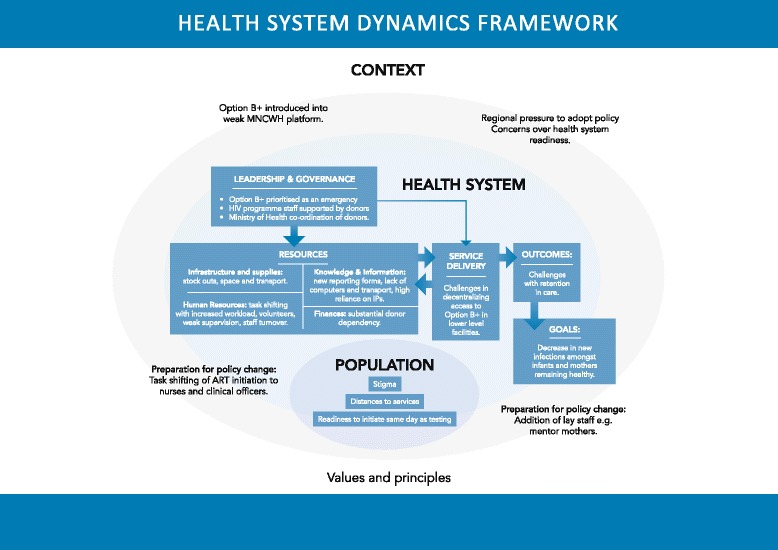
The Health System Dynamics framework adapted from Van Olmen et al. [20], populated with the findings of this rapid appraisal

We conducted a simple manifest analysis [23, 25], using these elements for grouping data into categories and themes. The analysis was based on the typed interviews, FGD transcripts, notes and reflections from the field. Results are reported in narrative form and presented according to the ten elements of the health systems dynamic framework. The van Olmen [20] framework has been adapted and populated with the findings from this study (Fig. 1).

## Results

**Context**: “*We can’t sit on the sideline. We have to move ahead and be more aggressive.”*

Adoption of Option B+ in Uganda, similar to several other countries in the region, was influenced by the international and regional contexts of rapid scale up of ART and by global HIV/AIDS commitments. Stakeholders described several compelling reasons to implement the policy shift. First, Uganda was seen as a PMTCT pioneer, as one of the countries where the first trial of Nevirapine was undertaken (HIVNET012 trial):

*“There was also… some regional pressure because … Uganda started PMTCT because we did the Nevirapine study here and people used to come to Uganda to learn about PMTCT and how to integrate it into the health system. But after a while we were lagging behind.” (Ministry of Health).*

Second, Malawi was a forerunner, adopting Option B+ prior to WHO endorsement of the guidelines, which prompted other sub-Saharan African countries to consider the policy shift:

*“I think when Malawi went to B+, and jumped in the deep end, people thought: they’re resource poor, their resources are even less than Uganda and they’re doing it, and why can’t we do it?” (Ministry of Health).*

Initially Uganda adopted a phased approach to rolling out Option B+, starting in 24 districts in October 2012. Following a technical review and funding commitments through PEPFAR, a decision was made to scale-up nationally, despite concerns amongst stakeholders around health system readiness:

*“At the time we were concerned that maybe we were trying to go too fast and that the country wasn’t ready to scale up to B+. But there was a lot of pressure from multiple angles to say: we can’t sit on the sideline, we have to move ahead and be more aggressive.” (Ministry of Health).*

Stakeholders also indicated that Option B+ was implemented within a primary health care platform that had to absorb additional PMTCT functions without broader health system investments:

*“PMTCT has been on the maternal health platforms which are largely primary health care …at levels of the healthcare system that were never designed to provide chronic care services, so that means the physical facilities, the staffing establishment was never designed to see the same patient coming in every day for the rest of their life. It was never designed to have progressively increasing numbers of individuals coming in for that service…and then you will see those effects in the quality of overall care that has been provided.” (International IP).*

**Population:** *“The woman came here with her luggage, she was kicked out.”*

At community level, Option B+ was implemented in a high-stigma environment. Mentor mothers, local mothers living with HIV and trained to counsel and support women newly enrolled in PMTCT, had to disguise the reasons for their home visits to avoid inadvertent disclosure of patients’ HIV status:

*“So either say that I am a sister or... they always give them some password. There are those that say please don’t come, and you know they have disclosure issues. We still have considerable amounts of stigma.” (Mentor Mother).*

A mentor mother shared the challenges of retaining women on lifelong treatment within the context of stigma and gender-based violence:

*“HIV is attached to infidelity, so when a man comes and tests positive he will think…he will know that the wife is the one who has brought HIV. Most of them fear because of gender-based violence. I tested a woman positive; I encouraged her to go and disclose…she went and disclosed. The husband chased her and told her “You go to the nurse who told you that you are HIV positive” and [in] the evening, the woman came here with her luggage. She was kicked out…she told me what can I do now? Where can I go?” (Mentor mother).*

Respondents indicated that the presence of peer support through mentor mothers and creation of family support groups helped reduce stigma in some communities:

*“Before a woman gets to the hospital they have someone at the village who knows (their HIV status). Previously it was uncommon for someone to stand up and say “I am HIV positive” because of the stigma attached. They will see no future in you; but now they are seeing HIV positive women who are empowered. We are positive and we stand up and tell people” “Yeah I’m positive, I am healthy, I am productive and I tell other women out there; so we are helping other people in communities to change their attitude.” (Mentor mother).*

*“The family support group has helped these mothers to take their drugs, to reduce some stigma, and it has also helped us in retention, and if you can see, all the babies are being discharged negative.” (Health worker).*

**Leadership and governance**: “*So all of us were left helpless, we were not getting any salaries”.*

Stakeholders at the national level described how shifting to Option B+ was implemented with a sense of urgency:

*“PMTCT was looked at as an emergency situation, you know, just to give it the right level of importance and attention. So, at the national level, every week, performance of every site of the country is reviewed.” (International IP).*

Stakeholders indicated that the role of multinational donors in supporting core oversight functions, including funding salaries and basic supplies, was critical:

*“Their (name of multilateral agency) support to the central, to Ministry of Health was really very important, because at that particular time between 2013 we had a bit of support from (name of GHI) but which was so critically on the PMTCT, most of the money was going through the different implementing partners in the region. So because (name of multilateral agency) came and had central Ministry of Health support, it was really helpful for us to be able to do proper oversight. Then between 2014 and now when there’s totally no (name of GHI) support to Ministry of Health, it has been I think the only fall back for Ministry of Health to be able to see what is happening in the other regions. It’s really the oversight, support supervision, mentorship, and you know, some things that we need within the office to do the oversight. Stationery, you know, that kind of thing.” (Ministry of Health).*

Another previous PMTCT focal person in the MoH described how his salary ceased when a major funding contract ended and how he left to work for an IP:

*“You know the challenges the Ministry of Health has, with all these positions which are supported by donors. Unfortunately, around February 2010 that agreement ended, so all of us were left helpless…we were not getting any salaries. So eventually they managed to transition a few of us including myself to government of Uganda payroll but the pay wasn’t attractive enough. So but I continued despite that till now June 2015 when this opportunity came, then I said let me give this a trial.” (Ministry of Health).*

Despite reliance on donor funding, respondents described the strong co-ordination role played by the MoH:

*“The Ministry people said, you know, as Ministry you are in charge of this programme. So I think that really helped. And so they came up and said, the partners will be in regions or in districts, and there will be preferably only one partner per district so that there is not a lot of overlap and confusion. I think that has helped.” (Ministry of Health).*

**Infrastructure and supplies:** *“How many times should she go back because of the health system?”*

Respondents across all levels mentioned shortages of supplies, especially HIV test kits. Health workers and managers used their own initiative to obtain ‘buffer’ stocks, through IPs or by borrowing from neighbouring facilities. Several respondents described the challenges stock outs pose to mothers, many travelling long distances:

*“For this last month, we had a stock out… we have just been borrowing, I mean getting from our near facilities. Then you have to rent a transport and then you bring, you cannot leave the mothers to go without.” (Clinical officer, Health Centre Level Three).*

*“Like the test kits, they are now out of stock. We are not testing mothers. Three weeks already there’s been no stock. Patient files, we don’t have. Even Septrin 480 (co-trimoxazole), is out of stock.” (Mentor mother).*

*“For me I think, I know okay, one supply thing that affects the programme is HIV testing and counselling kits. You mobilise women, they never get tested and they go back to the community. The community mentor mothers and village health teams (VHTs) will do everything they can to get them back, but the lady’s using transport, she’s walking…. I mean how many times should she go back because of the health system?” (International IP).*

Respondents also spoke of infrastructural challenges: insufficient physical space in facilities and lack of transport for outreach activities:

*“If we go on with the way we are taking course, we shall require to have a very bigger space. Because this one was supposed to be first stay room for labour; the other one second stay, but because of space we have turned it into an ART clinic now.” (Health worker in charge, health centre level three).*

*“So now we said, okay, we’ll make use of those cars since the other project closed. But by the time we really took off with our activities, we found so many of these cars had no tyres, or broken engines. So we couldn’t reach all these planned sites. Now we had just about 48% of the planned outreaches done last year, 2014…. I mean, [it was] nice in the way that, sometimes in the middle of nowhere, I mean, you just see this mass of children and women that previously, if they had to get that service, would have to travel maybe 50 kilometres.” (International IP).*

**Human Resources:** *“But if you want these people (volunteer community workers), somebody is wasting his time, which he would have used to plant food in his home.”*

In preparation for Option B+, two main activities took place. These were task-shifting and capacity-building of nurses/midwives to initiate, manage and contribute to surveillance of women on lifelong ART, and recruitment of community cadres to assist nurses with counselling and adherence support. Additional tasks for nurses did not come with additional posts, and several respondents described their workload increase:

*“Adjustments we had to make in the country is to expand the initiation of antiretrovirals (ARVs) to non-doctors, so we had to train and allow the clinical officers and the nurses and the midwives to initiate.” (Technical officer, multilateral agency).*

*“Now the staffing norm, when you seeing crowds building up on a monthly basis, because we keep getting a rise in numbers every month…if you consider the deliveries, we started off at 80-something [per month],… now we are at around 140, still the same staffing levels. Then you have the HIV clinic, the ART clinic, the mother-baby care point, you keep creating small services…you are spreading the staff thin.” (Health worker in charge, Level 4 health centre).*

The challenge of ongoing training due to staff turnover and rotation, combined with maintaining facility staffs’ clinical skills, was expressed by the IPs who supported the initial training and continue to provide facility supervision:

*“Another challenge we were getting was that some of the health workers that are trained were being transferred to other health units that were probably not [offering Option] B+. And you find that you go to a facility that you already knew was implementing Option B+. Before you get there, the person who you are training is no longer there. So you have to either do on-site mentorships for probably a new person they have brought in, who might not have been trained, so it was a bit of a challenge.” (International IP).*

*“I know ART has come a long way and it can be provided by a whole host of healthcare workers, but one still needs decent clinical care expertise. Several times, when our technical staff are out at a district or a site, they will be the most skilled person the site is going to see for the next maybe six or eight weeks, until they come back.” (International IP).*

Most community cadres recruited for counselling and retention support are volunteers, except within the Mothers2Mothers model. One lay counsellor described how they help relieve nurses’ workload, but struggle to generate income for their own livelihoods, so they forego volunteer activities when paid work is available:

*“Since we are volunteers at this clinic, you find that we can’t come every day. We come two days in a week or three, so you find that at least they get actually a burden of work on other days when we are not there. And if you find a small job to work [for] some money, you find that you have missed some for two weeks, hey? And it is a challenge somewhere at the clinic here.” (Lay counsellor).*

*“Every person has problems, but if you want these people, somebody is wasting his time which he would have used to plant food in his home. But he’s doing some good work in the community and bringing community mobilization … bringing community referral to health facilities… making linkages. And [he] is actually not given even a bar of soap at the end of the month. I look at it as being inhuman.” (District ART and PMTCT focal person).*

**Knowledge and information: “** *To tell you the truth, without support from the implementing partners, very little will happen at district level in terms of getting that data in the system.”*

Introduction of Option B+ created additional data reporting requirements. Challenges included lack of computer equipment for data-entry within facilities, requiring either district biostatisticians to travel monthly to collect forms or facility managers to bring the data to the district office. This was hampered by shortages of transport and reliance on allowances from IPs. As several managerial respondents described:

*“Most of these IPs have been the ones that are printing the monitoring and evaluation tools. So first, a challenge that there are times when you get stock outs of the registers and other monitoring tools. And… government can’t support us. Like right now they are rolling out the new health management information system; but they are rolling it out with no registers at the facilities. So you only have to wait for the IPs to help us. And yet for them, they want us to report according to the new reporting tools, hey?” (Health worker, level 4 health centre).*

*“To tell you the truth, without support from the implementing partners, very little will happen at district level in terms of getting that data in the system. Depending on how much resources and other technical support the IP has put in. If this biostatistician is not given an allowance to actually see it, and enter this data, then it will not happen.” (Multilateral agency).*

**Finances: **“*So if donors woke up tomorrow..., and said that we can’t fund you… what would we do?”*

Financial sustainability of Option B+ was a recurring theme. Several respondents described the programme vulnerability, should funding reduce. The addition of community cadres was greatly appreciated by health workers, although lack of sustainability was reported as a major threat:

*“Yeah we have them [mentor mothers]. But I think their contracts are…they are supposed to expire …just disaster now. What their input has been doing in this community has been really great. And their failure to renew means we are just going back to where we were in the past.” (Health worker, level 3 health centre).*

*“I give you a simple example of the peers, I think the peers had three months without support. And they are only getting like 80,000 (US$22) per month, but when that money stopped, most of them stopped going to the facilities. And you wouldn’t blame them. So it is, at times, very difficult to run these structures without any support.” (District health officer).*

*“So 90% of the funding for Option B+, maybe 95%, actually is donor dependent. So if donors woke up tomorrow..., and said that we can’t fund you… what would we do?*” *(Anonymous interviewee).*

Several respondents expressed frustration that major donors funded consumables, but not the human resources needed to support retention, such as the community cadres and VHTs:

*“Right now, as we are going to the future, (name of GHI) is more and more meeting costs for the big ticket items, the medicine. So, there are gaps with the softer things which are so critical. So, we are doing rapid initiation of ARV in individuals. These women are healthy, they are young and we are starting them on lifelong treatment very quickly. So, you know, one has to prepare them.” (International IP).*

**Service delivery**: “*There are buildings but there are no health workers, so we can’t initiate.”*

The major challenge regarding service delivery was decentralisation of Option B+ services to lower level facilities (level 2 and 3 health centres), with shortages of human resources and lack of physical infrastructure:

*“Our wish is to make sure that all the Health Centre 2s are currently implementing these services, but there are some Health Centre 2s that are white elephants. In other words, there are buildings but there are no health workers, so we can’t initiate. So, if you look at our 3118 health centre 2s that we have in the country now…. Almost all are not implementing Option B+ because of either human [resource] constraints, the others do not have healthcare workers to be trained, the others do not have space because some of them are just one block, which is just maybe the reception and the OPD and one treatment room. There is no lab. So now what we are trying to do with the IPs is, we are trying to identify health centre 2s which are handling a high volume and try to see, can we help them create room for privacy, for space, for counselling and once that is done, we can then introduce the services.” (Ministry of Health).*

*“We have health centre 2s, which are delivering mothers, but they are not carrying out HIV testing, so in that way, we are having missed opportunities. The nearest testing site is far and some of them don’t want to travel that long distance when they are referred there.” (District health officer).*

**Outcomes and Goals:** “*Our challenge is retaining them on treatment”.*

Option B+ policy goals are: to achieve universal coverage of testing and ART for HIV-infected women; to ensure that women remain in care; to ensure elimination of new HIV infections among children; and keeping their mothers alive. Respondents indicated that although coverage of testing and ART initiation improved since Option B+ policy adoption, retention in care remained a challenge:

*“Our biggest challenge now we are grappling with, is keeping these women in care. We have done well, in Uganda now, every eight out of ten mothers who are pregnant and lactating women with HIV are on drugs. So, we have done well in initiating them on drugs. Identifying them … testing them: we are doing well. We still have high stigma and discrimination in our country, so these high levels of stigma is still causing treatment interruptions.” (Ministry of Health).*

*“We need people in the community to track them…because the VHTs [Village Health Teams] have long distances, there’s a limit as to how much they can go for free. Yeah. So that’s, to me, a weakness in PMTCT for the whole country. And there again, of course, it also falls back into tracking the mothers, because I know as a country we are not doing well in terms of retention.*” *(International IP).*

Respondents described reduction in new HIV infections amongst children as a major success of Option B+:

*“Okay. We’ve realised that positivity rate is somehow going down because at first…, before B+, we were like at 10.5%, but now we are at 6.8% and we hope to improve.” (District hospital PMTCT focal person).*

*“Our most success is having babies who are HIV-free. And even seeing that our mothers are living a healthy life. And their families as well.” (Mentor mother).*

## Discussion

This qualitative study uses the health system dynamics framework [20] to explore the impacts of the scale-up of Option B+ on the health system in Uganda. Our findings reveal that contextual pressures (ambitious global targets to eliminate new HIV infections, a bold shift to a lifelong ART programme in Malawi and rapid global guideline change) all compelled Uganda to shift to Option B+ soon after the WHO guidelines were released.

Our findings revealed that senior stakeholders held concerns about the health system’s readiness to adopt this policy and about the rapid pace of scale-up. At the international level, concern was raised by public health experts soon after the WHO programmatic guidance was released [26], yet focus remained on clinical goals with little attention to health system readiness. Authors from the International Community of Women Living with HIV argued recently that rapid implementation of Option B+, without investing in community-based responses and ensuring universal access to quality healthcare, is tantamount to neglect of women’s human rights and dignity [27]. A recent cross-sectional assessment of health care facilities to assess roll-out of Option B+ in Uganda confirms our qualitative findings. The assessment found that less than a third of level 3 health centres performed routine diagnostics, including syphilis, hemoglobin, and urinalysis testing which are basic requirements at all ante-natal clinics [28].

Respondents revealed that the scale-up of Option B+ impacts on service provision through its effects on several elements of the health system: human resources, infrastructure and supplies, routine health information and finances. Implementation of Option B+ has been plagued by stock-outs of basic supplies (HIV test kits and ARVs), forcing health workers to borrow from neighbouring facilities to minimise service interruptions. Though the Global Fund has disbursed over $400 million since 2003 to support the HIV/AIDS response in Uganda, a report from a 2015 audit of Global Fund investments in Uganda revealed that 70% of health facilities audited reported stock-outs of ARV medicines and HIV test kits lasting between 3 weeks and 4 months [29].

Lack of transport for supervision and insufficient physical space in facilities also hampered service provision, including outreach activities. Whilst task-shifting increased access to ARVs, it also added to the workload of already overburdened nurses. Lay cadres were recruited to assist with counselling and retention monitoring, although these cadres were mostly volunteers, having to maintain activities to support their own livelihood. Consequently, within a context of weak infrastructure and human resource shortages, incomplete decentralisation of Option B+ meant that services were not sufficiently accessible to some communities.

Respondents described how the shift to Option B+ was managed as an emergency, with close monitoring of district performance. Whilst the MoH played a strong co-ordination role, it was heavily reliant on multilateral donors and IPs for national HIV programme staff salaries and stipends, capacity development, commodities, routine health information and clinical supervision. Furthermore, the consistency of these activities was highly dependent on donor funding. There is increasing evidence of unintended consequences of GHIs, particularly those focussing on HIV/AIDS control, on routine health systems [13, 16–18, 30]. A recent assessment of the impact of PEPFAR investments on health systems strengthening in Uganda found declines in utilisation of non-HIV care in medium and high ART-investment districts. This was apparently due to the ‘emergency’ nature of the investments, restricted to vertical HIV/AIDS-related activities, with resultant diversion of scarce human resources from other non-HIV services [13].

This study has highlighted that critical elements of the health system required to implement Option B+ were dependent on external donor funding. This is a common feature of the HIV/AIDS response across sub-Saharan Africa with the exception of South Africa and Botswana, and highlights the imperative for increased domestic funding. Taking Uganda as an example, funding for the current Ugandan National HIV and AIDS Strategic Plan (NSP) (2015/2016 to 2019/2020) is projected to require US$ 3647 billion. The NSP has an estimated financing gap of US $918 million by 2019/2020, assuming domestic funding rises to 40% of NSP requirements. Currently it is at 12% [31]. In order to meet the growing need of increased patient numbers and increased ART requirements under the UTT policy, the projected costs of comprehensive HIV care and treatment services in Uganda will increase substantially in the future. Furthermore, researchers have argued that financing strategies should also take into account health systems strengthening requirements [32].

Despite the large investments in the HIV/AIDS programme in Uganda, in 2014, only 50% of adults and children living with HIV were receiving ART [3]. There is growing recognition that weaknesses in health systems contribute to a failure to improve health outcomes in low and middle income countries, despite increased official development assistance [33]. Health systems face the repeated intrusion of new policies, programmes and initiatives, some of which have overlapping goals. This can be disruptive and compete for the time and attention of health workers and managers, rather than reinforcing an integrated service delivery platform. However, there have been few reported sustained attempts at health systems strengthening across programme areas. This is an area in urgent need of implementation research, as health system strengthening should not be done for one programme area alone. Furthermore, it is not a once-off process but requires long term commitment through continued investment and co-ordinated planning. Whilst we use Uganda as an example in this paper, there is an imperative to strengthen health systems across the region, to enable greater resilience in the face of a rapidly changing health policy and implementation context.

### Limitations

A limitation of this study is the rapid appraisal approach to field research during a short country visit. The impressions presented must therefore be regarded as snapshots, raising questions for further exploration. Furthermore, the evaluation terms of reference explicitly excluded interviews with clients (beneficiaries of services). However, a strength of the study is the wide range of participants, from senior national managers to community cadres, across all regions of the country.

A key strength of the health system dynamics framework is that it facilitates analysis of different health system interfaces: population-health system interactions, organisation of service delivery (resources), and interactions between service delivery and the broader context, which impacts on goals and outcomes.

## Conclusion

Uganda has achieved success in scaling up access to ART and reducing the number of children newly infected with HIV, from 24,000 in 2000 to 3500 in 2015 [34]. Sustaining these gains may be threatened by health system weaknesses.

This study uses the health system dynamics framework as an analytic approach to explore the effects of new policy implementation on critical elements of the health system. Its emphasis on relationships between system elements, population and context is helpful in unpacking impacts of and reactions to additional pressure on the health system, which adds value beyond some previous frameworks.

As Uganda scales up its UTT policy in 2017, lessons from the scale-up of Option B+ should carefully be considered. Approaches such as benchmark tools, which have been applied to assess health system readiness to scale-up newborn survival interventions [35], could be applied to national HIV/AIDS interventions with specific actions and lines of accountability for addressing benchmarks not met, prior to scale-up and ongoing monitoring of health system performance.

If ongoing investments and technical support for the HIV/AIDS response in Uganda are not explicitly directed to ‘spill over’ into support for strengthening the health system across programme areas, a significant opportunity may be lost.
